# The cost-effectiveness of population Health Checks: have the NHS Health Checks been unfairly maligned?

**DOI:** 10.1007/s10389-017-0801-8

**Published:** 2017-04-21

**Authors:** Sebastian Hinde, Laura Bojke, Gerry Richardson, Lise Retat, Laura Webber

**Affiliations:** 1 Centre for Health Economics, University of York, Heslington, UK; 2 UK Health Forum, London, UK

**Keywords:** Economic evaluation, NHS Health Checks, Vascular health, Obesity

## Abstract

**Aim:**

The English NHS currently has a policy of providing Health Checks to all 40–74 year olds. Administered in primary care, they aim to identify patients at risk of a range of diseases, including diabetes and heart disease, and facilitate care. This study is the first to use observed data on the effectiveness of the Checks to consider whether they represent a cost-effective use of limited NHS resources.

**Subject and methods:**

Using a publicly available evaluation tool we conducted an analysis of the Checks to establish the long-term cost and health-related outcomes of a cohort of patients. The primary focus of the analysis was to establish whether the impact of the Checks on BMI was sufficient to justify their cost.

**Results:**

The Checks were associated with a reduction in mean BMI of 0.27 (95% CI 0.20 to 0.34) compared to no Check. When applied to the evaluative tool, a small but positive QALY gain of 0.05 per participant was observed, coupled with a reduction in disease-related care costs of £170 ($210 USD). When the estimated cost per Check (£179, $220 USD) is taken into account, we estimate an incremental cost-effectiveness ratio of £900/QALY ($1109 USD/QALY).

**Conclusions:**

Much of the criticism of the Health Checks has focussed on the relatively small average change in risk factors such as BMI. However, this analysis suggests that the significant health and cost-saving benefits from even a modest reduction in mean BMI, coupled with the low costs of the Checks, combine to result in a potentially highly cost-effective policy.

**Electronic supplementary material:**

The online version of this article (doi:10.1007/s10389-017-0801-8) contains supplementary material, which is available to authorized users.

## Introduction

Since its initiation in 2009, the policy of providing NHS Health Checks to 40–74 year olds every 5 years with the aim of reducing the risk factors associated with heart disease, stroke, type 2 diabetes, kidney disease and some types of dementia has proved controversial (Abdalrahman and Soljak 2015). Despite the Department of Health’s initial, hypothetical, cost-effectiveness analysis suggesting the policy was highly cost-effective, recent independent research has shown both a poor level of coverage (Robson et al. 2016) and overall programme performance substantially below initial targets in terms of reduction in cardiovascular disease risk factors (Chang et al. 2016).

The impact of this contradictory research has been compounded by the shift of commissioning responsibility for the policy to Local Authorities. These bodies have experienced budget cuts of more than one third since 2010, which have been unevenly distributed with consequent inequalities in funding capacity (Innes and Tetlow 2015). While the Health and Social Care Act of 2012, which instigated this shift, obligated Local Authorities to provide a number of services including some form of population Health Checks (Heath 2014), a number of Authorities are looking to meet this requirement through a more targeted (risk profile) approach in an attempt to reduce their cost expenditure—a response that undermines the principle of equity that underpinned the initial aim of the Health Checks.

Within the context of this shift towards disinvestment, this article provides an evidence-based cost-effectiveness evaluation of the NHS Health Checks. We analysed the impact of the policy in terms of reducing the long-term risk factors associated with obesity, one of the primary drivers of many of the diseases targeted through the Checks. This focussed analysis seeks to answer the question: has the reduction in obesity brought about by NHS Health Checks been sufficient to justify the cost of the entire policy to the NHS? We employed a publicly available economic evaluation toolkit (EConDA http://www.econdaproject.eu/tools.php) to estimate how an observed reduction in the mean BMI in the target population can be expected to affect long-term population health and costs to the NHS.

In light of Local Authorities increasingly focusing on a targeted programme of Health Checks and the National funding of the new, targeted Diabetes Prevention Programme, “Healthier You”, a secondary analysis was conducted to explore whether such a targeted approach can be expected to be a more cost-effective means of achieving a population health improvement.

## Previous evaluations of the NHS Health Checks

Prior to launching, the cost-effectiveness of the NHS Health Checks initiative was evaluated by the Department of Health (Department of Health 2008), which estimated an incremental cost-effectiveness ratio of £3000/QALY ($3690 USD/QALY), well within the NICE cost-effectiveness threshold for public health (NICE 2012). However, this estimate has been heavily criticised, with several authors pointing to the unrealised assumption that the initiative would achieve 75% uptake and suggesting that uptake may be closer to 20% (DTB 2016). However, since most of the costs for the Health Checks occur at the point of uptake, it is not clear that a reduction in uptake would have a significant impact on the policy’s cost-effectiveness at a per-patient level since if fewer Checks are conducted, the policy will be both less effective and less expensive.

Further criticism of the evidence underpinning the Health Checks came in October 2014, when the House of Commons Science and Technology Committee Report on National Health Screening was published. This criticised the introduction of national NHS Health Checks: ‘without an evidence base demonstrating that it could achieve its aims and we are concerned that it could be, as a result, wasting resources’, p. 45 (Science and Technology Committee 2014). The Committee recommended a retrospective evaluation of the programme by the UK National Screening Committee. In their response, the Government pointed to the 2013 report by Public Health England (Department of Health 2015), which outlined the evidence base and highlighted the then ongoing research published by Robson (Robson et al. 2016) and Chang (Chang et al. 2016).

While much criticism of the NHS Health Checks has been published, only limited research has been conducted on the cost-effectiveness of any form of population Health Checks whatsoever. The most relevant example (an evaluation by Schuetz et al. concentrating on vascular health alone, and as such a more focussed policy than the NHS Health Checks) suggests that such Checks can be cost-effective (Schuetz et al. 2013). Schuetz uses a number of theoretical scenarios to investigate the cost-effectiveness of vascular Health Checks across six European countries, including the UK, finding that for all scenarios considered beyond a 10-year time horizon of evaluation, Checks in the UK were cost-effective. However, the hypothetical nature of the effectiveness data used reduces the relevance of these results to UK policy makers.

## An economic evaluation of the observed impact of the Health Checks on vascular health

As highlighted in the previous section, only two published economic evaluations have been identified that consider the cost-effectiveness of population Health Checks in a UK setting (Department of Health 2008, Schuetz et al. 2013). However, both relied on hypothetical estimates of the efficacy of the policies. This section presents the results of an exploratory economic evaluation, using recently published estimates of the impact of the Health Checks applied to a publicly available economic modelling toolkit.

### Vehicle to link estimates of effect to costs and outcomes

The starting point of this analysis was the identification of a previously constructed economic evaluation model. To enable any future users of such an analysis, including local authorities, to generate results in a timely fashion using local level data, the ideal model was defined as being a publicly available economic evaluation of the disease set targeted by the Health Checks, applicable to a UK setting. While no models could be found that considered the full Health Check disease set, a number of models were identified and deemed to have sufficient links to the aims of the Health Checks to provide a robust evaluative framework.

The EConDA toolkit (http://www.econdaproject.eu/tools.php) was selected for this evaluation because of its strong predictive abilities in relation to the expected trend in obesity-related disease without policy intervention and its strong health economics component, considering both long-term costs and quality adjusted life years (QALYs). The tool is based on three assumptions: First, it is deterministic. The main method is to calculate an individual’s risk of getting a disease based on their age, sex, current disease state, medical history and risk factor level. This probability is included in a life-disease table that estimates the probabilities of being alive with no disease. Second, the deterministic tool processes cohorts made up of weighted individuals where the weight is calculated as shown in the following equation:

$$ cohort\  member\  weight\ \left[ i, j, k, l\right]={p}_{sex}(i)\times {p}_{age}\left( j| i\right)\times {p}_{rf}\left( k| i, j\right) $$

$$ whereby: i\in \left[0,1\right], j\in \left[0, n\right], k\in \left[0,2\right] $$

- *p*_*sex*_ *(i) is the probability of being male or female*
- *p*_*age*_ *(j|i) is the probability of having a certain age given sex*
- *p*_*rf*_ *(k|i, j) is the probability of being in a certain category (*i.e. *smoker, non-smoker, normal weight, overweight, obese) given sex and age.*

Finally, the toolkit is structured around the estimation of the prevalence of four obesity-related diseases [chronic heart disease (CHD), hypertension, stroke and diabetes] in a cohort of interest, with and without an intervention.

While this set of diseases does not perfectly accord with those targeted by the Health Checks (which look at CHD, stroke, diabetes, kidney disease and dementia), the focus of Health Checks on providing support in reducing the rate of obesity is a primary element of the intervention. Furthermore, an economic evaluation of kidney disease and dementia (the only two not considered in the EConDA tool) would be highly challenging because of the poor definition of what constitutes kidney disease and the challenges associated with the economic evaluation of mental health issues such as dementia (Evers et al. 2007).

### Estimating the impact of the NHS Health Checks

To obtain information about the effect of the NHS Health Checks on population health, a rapid review of the literature was conducted to find evidence of impact. Only two studies fulfilled the criteria.

First, Forster et al. (2015) followed a cohort of 140,356 patients in the UK who underwent a Health Check in 2012. Follow-up data were available for a sub-set of patients at 15 months (*n* = 52,385). The mean reduction in BMI over this period was −0.28 (95% CI −0.23 to −0.32) BMI points in men and −0.19 (95% CI −0.15 to −0.24) in women. No control population was included in the analysis.

Chang et al. (2016) conducted a retrospective analysis of 138,788 patients aged 40–74 years registered with 462 English general practises participating in the Clinical Practice Research Datalink (CPRD) between 2009 and 2013, including those who had, and had not, attended the Health Checks. They applied a difference-in-difference matching methodology to compare changes in a number of outcomes between the two groups including mean BMI, with a median follow-up of 2 years. The change they found in mean BMI as a result of the Health Checks was similar to that found by Forster et al. (−0.27 with a 95% CI of −0.20 to −0.34). However, in contrast to Forster, this difference was driven by an increase in the mean BMI of non-attendees (0.30 95% CI 0.29 to 0.30) rather than a decrease in that of attendees (who saw a small mean increase of 0.01, 95% CI 0.00 to 0.02).

While it is unclear why the two analyses showed similar relative changes in mean BMI but different absolute changes in the mean BMI of the population attending the Health Checks (−0.28 and −0.19 BMI point reductions for men and women in Forster, but a 0.01 point increase in Chang), the inclusion of a control population in Chang makes it methodologically more robust (Dimick and Ryan 2014). Therefore, we only carried forward the relative reduction in mean BMI observed in Chang.

### Estimating the per-patient cost of Health Checks

Finally, an estimate of the per-participant cost of the Health Checks is required, including the cost of follow-up interventions such as smoking cessation and weight management support. The original Department of Health report (Department of Health 2008) estimated the total annual cost of providing the Checks (£36 million, $44.3 million USD) and the resulting interventions (£161.1 million, $198.2 million USD) to a population of 1.1 million (based on 3 million eligible per year, 2.2 million participating and 1.1 million already receiving some form of vascular check). While, as discussed above, the rate of patient enrolment was less than originally projected, most of the costs occur at the point of conducting the Check, meaning that the cost per participant is not assumed to be impacted by the level of participation. The total cost of the Checks is therefore £197.1 million (£242.4 million USD), an estimated £179 ($220 USD) per Check conducted, assuming no cost is incurred by those who are eligible but do not attend a Check. Importantly, this estimate includes the cost of interventions directly resulting from the Checks, but not the long-term cost of care implications, which are already incorporated into the EConDA tool. An upper and lower estimate of the total cost of £243 million (£221 per Check, $272 USD) and £180 million (£163 per Check, $200 USD) was provided in the Department of Health analysis and used to inform scenario analyses in this evaluation.

## Results

The evidence collected in the previous section was used to inform an analysis of the cost-effectiveness of Health Checks compared to no intervention. Taking into consideration the costs to the NHS and health benefits to patients across a lifetime, the details of the analysis are presented in Appendix 1.

Based purely on the population health gains resulting from the change in BMI observed by Chang et al. (i.e. considering the full cost of the Health Checks but none of the population health benefits associated with non-weight related illness), Health Checks were shown to reduce the prevalence of all four diseases modelled by the EConDA tool, with peak prevalence reduced by 2.1% for CHD, 1.6% for diabetes, 1.5% for stroke and 0.8% for hypertension.

This resulted in the Health Checks being highly cost-effective, associated with an incremental cost effectiveness ratio (ICER) of £900/QALY ($1107 USD), an estimate of the additional cost per QALY gained as a result of implementing the Health Checks. This result stems from the small incremental cost associated with the Health Checks [the combination of a low average cost per participant attending (£179, $220 USD) and cost savings due to reduced disease-related care in the long term (average saving of £170 per attending patient, $209 USD)] and the small QALY gains (an average of 0.01 QALYs per person attending the Checks).

The analysis necessitated a number of simplifying assumptions due to the available evidence and structure of the EConDA toolkit. In addition, a number of the scenarios considered in Appendix 1 found the Health Checks to be potentially more expensive but no more effective than a total lack of intervention. However, the results highlight that, because of the relatively low cost of the Checks per attendee [estimated at £179 ($220 USD) including follow-up services where applicable], the potential cost savings due to reduced future treatment of diseases (an average cost saving of £170, $209 USD) and the unobtrusive nature of the Checks and subsequent tier 2 weight loss services (resulting in no detrimental health effects), the Checks are highly likely to be cost-effective if any beneficial impact on the outcomes of interest can be shown.

## Targeted diabetes programme

An alternative to the broad-spectrum approach of the Health Checks is a targeted, disease-specific approach. While no direct, evidence-based, comparative evaluation has been conducted, Schuetz et al. (Schuetz et al. 2013) argued in their hypothetical evaluation that a targeted approach to Health Checks based on pre-screening patients by age, vascular risk score and BMI dramatically improves cost-effectiveness, even making the intervention dominant over a policy of no Checks (i.e. more effective and less expensive). The intrinsic appeal of such a targeted approach comes from its potential to reduce costs compared to a broad-spectrum approach, while maintaining overall effectiveness by only conducting a Check on patients with a high propensity to benefit.

NHS England recently launched such a programme, seeking to reduce the prevalence of risk factors associated with diabetes through the Healthier You: NHS Diabetes Prevention Programme (Triggle 2016). No evaluation of the expected clinical or cost-effectiveness of the Healthier You programme appears to have been conducted prior to commissioning.

The new targeted programme aims to target 20,000 patients at risk of type 2 diabetes in its first year (2016), at a cost of £7 million ($8.6 million USD), equating to £350 ($431 USD) per patient. The programme will offer 13 sessions of healthy lifestyle support to people identified by their GP as being most likely to benefit. By 2020, 100,000 places will be available per annum. This number is comparable to the 200,000 new cases of type-2 diabetes diagnosed each year in England.

A body of literature exists evaluating the cost-effectiveness of type 2 diabetes prevention activities, with most of it finding prevention through intensive lifestyle changes and/or pharmacological intervention to be cost-effective, and often cost-saving (International Diabetes Foundation 2015). However, the significant variation in the nature of each intervention, reflected in factors such as its structure and the population targeted, has led to a significant degree of variation in their cost-effectiveness. As such, only limited inference can be drawn from the existing literature as to the cost-effectiveness of the Healthier You programme, which would require a programme-specific evaluation to determine whether it represents a cost-effective use of limited NHS resources. The following estimates bring together existing evidence on the Healthier You programme to consider the impact required to demonstrate cost-effectiveness. The EConDA toolkit was used to gauge the change in the level of obesity (the primary type 2 diabetes risk factor targeted by the programme) necessary for the programme to be cost-effective.

Assuming a threshold of £20,000/QALY ($24,600 USD/QALY), in order for the programme to be cost-effective (£350 per person, $430.5 USD), it would need to be associated with a QALY gain of 0.0175 QALYs (£350/£20,000) per patient. If the programme resulted in additional cost savings due to reduced future treatment costs borne by the NHS, and these exceeded the initial £350 ($430.5 USD) per participant programme costs, it could be said to dominate a policy of no intervention. This would save the NHS money in the long term and improve population health.

**Fig. 1:**
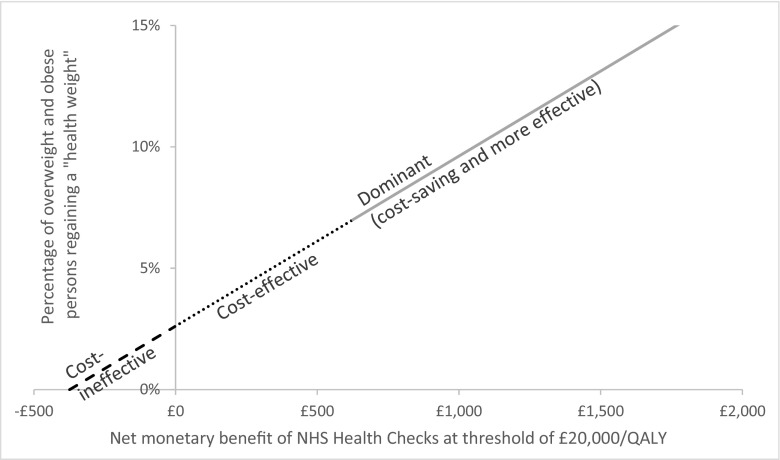
Impact of effectiveness of intervention on potential for cost-effectiveness

As no estimate of changes in obesity levels resulting from the targeted programme exists, our analysis sought to determine the point at which the policy could be considered cost-effective or dominant compared to a policy of no intervention. All models were run for an “at risk” 18+ cohort to reflect the targeted nature of the policy, with no age restrictions and a lifetime analysis horizon. Figure 1 shows the results of this analysis, plotting the percentage of participants returning to a healthy weight (BMI of between 18.5 and 25.0) against average cost savings from reduced future care.

The figure shows that a linear relationship exists between the effectiveness of the Healthier You programme and the potential for cost-effectiveness (represented by the net monetary benefit of the programme at a cost-effectiveness threshold of £20,000/QALY, $24,600 USD/QALY). The analysis suggests that if the Healthier You programme resulted in less than 2.6% of participants reaching a healthy weight, it would represent a cost-ineffective use of limited NHS resources since the cost of intervention is more than the combined net health gain and cost-savings of reduced future treatment. An effect of between 2.5 and 7.0% would result in the Healthier You intervention being a cost-effective use of NHS resources since the net health gains would then be greater than the cost of the intervention. For all levels of effectiveness greater than 7.0% the policy dominates a policy of no intervention since the cost savings from reduced future obesity-related disease treatment becomes greater than the £350 ($430.5 USD) per participant cost of the Healthier You programme.

## Discussion

Two prevention techniques are currently being undertaken in the UK to address the problem of a rising number of deaths from cardiovascular disease and the increasing incidence of diabetes. These are the NHS Health Checks and the recently initiated Healthier You programme. While the underlying aim of these programmes is similar, with both using low-tier primary care interventions to reduce the risk profile of patients, the methods used are very different. The NHS Health Checks have taken a broad approach to both the diseases of focus and their associated risk profiles and the recruitment criteria. By contrast, Healthier You only seeks to reduce type 2 diabetes risk profiles in targeted patients. In addition, the Healthy You programme has a targeted approach to recruitment, with GPs identifying those who would most benefit, whereas age is the only criteria applied for recruitment to the Health Checks. Finally, it also incorporates a programme of healthy lifestyle support, in contrast to the Health Checks, which only rely on existing advice and NHS programmes to reduce the risk factors of interest.

Given current evidence, it is impossible to determine the impact of the programmes’ respective features on their relative effectiveness, making a meaningful comparison of the two impracticable. While a targeted approach (both in disease and population) may result in a more efficient service by only enrolling patients who will/may benefit, it may, by being too narrow, risk missing patients who may also stand to benefit.

Additionally, it is unclear whether the Health Checks and Healthier You programmes are considered substitutes or complementary programmes at a national or local level. While in the current funding environment it is unusual to have two public health programmes addressing the same complex, the scale of the obesity and diabetes problem facing the NHS may justify two such programmes existing in tandem.

This study evaluated the potential for the two programmes to be independently cost-effective. Despite significant criticism of and disinvestment in the Health Checks, we were unable to find robust evidence in the literature supporting a lack of cost-effectiveness. Our focussed evaluation considered whether the observed change in BMI due to Health Checks was sufficient to demonstrate cost-effectiveness in isolation from the other aims of the programme. The analysis found that, despite the small change in BMI observed, due to the small outlay and potential for large QALY gain and cost savings from reduced disease treatment expenditure, cost-effectiveness was likely.

Similarly, the recent roll-out of the Healthier You programme does not appear to have been accompanied by an economic assessment prior to investment. However, our exploratory evaluation suggests that the required impact of the programme in reducing the diabetes risk profile of participants is small, needing just 2.5% of participants to gain a healthy weight for the programme to be a cost-effective use of limited NHS resources.

While our appraisal seems to show potential for both programmes to be cost-effective, there are a number of factors that cannot be overlooked. First, as is typical of preventive interventions, the immediate cost is paid now, while the benefits in terms of population health and savings occur in the distant future. As a result, even if strong evidence of cost-effectiveness was demonstrated, Local Authorities experiencing financial restraints and under pressure to meet short-term targets may still choose to not invest in such programmes.

Second, the evaluations presented here are based on little or no data and significant assumptions. While given existing evidence the analyses represent the most robust approach available, significant research is still required. For example, our analysis of Healthier You concludes that a relatively small proportion of participants would need to regain a healthy weight for the programme to be cost-effective or dominant. However, no evidence exists as to whether this impact is achievable with the programme despite its relatively small scale. Furthermore, this study focussed on a single risk factor, BMI. Future work should consider the impact of Health Checks on multiple risk factors simultaneously, such as BMI, smoking and alcohol consumption.

Finally, while the NHS Health Checks may be cost-effective in their current form, it is highly likely that through improved links with cost-effective treatment programmes, and the use of self-management initiatives, such as user-operated “Health Kiosks” to conduct screening, the effectiveness of the programme could be improved and costs reduced.

## Conclusion

This article has updated the debate around the cost-effectiveness of policies seeking to improve population health through primary care interaction. Contemporary evidence on the impact of NHS Health Checks on the mean BMI of participants suggests that the policy’s impact on reducing obesity-related disease factors may be sufficient to demonstrate that it is a cost-effective use of limited NHS resources. Similarly, the new Healthier You initiative has the potential to be cost-effective if relatively small improvements in the levels of obesity can be achieved. In both cases, the low per-participant cost of the programmes and negligible adverse events, but potentially large gains in future population health and NHS savings are the driving force behind the potential cost-effectiveness.

Our analyses show that relying on studies that examine the short-term effectiveness of such policies alone can be misleading. Without full consideration of the long-term implications to population health and NHS resources, the potential for such policies to be cost-effective may be overlooked. However, a full assessment is required, which not only evaluates the merits of the programmes as they exist but also the full range of potential designs of intervention, disease inclusion and populations covered. While such an appraisal would be methodologically challenging because of numerous potential combinations, existing methods of economic evaluation and information analysis would be able to guide decision-makers as to the optimal design of any intervention. As the analyses presented show, the potential exists for large gains in population health and cost savings to the NHS. Despite this, the investment and disinvestment in programmes such as the NHS Health Checks and Healthier You appear to be based on little to no evidence. As a result, money is being wasted treating preventable diseases and lives are being lost.

## Electronic supplementary material

ESM 1 (DOCX 365 kb)
