# Supplementary material for: The cost-effectiveness of population Health Checks: have the NHS Health Checks been unfairly maligned?
Source: Z Gesundh Wiss. 2017 Apr 21;25(4):425–31. doi: 10.1007/s10389-017-0801-8 (PMC5515950; doi:10.1007/s10389-017-0801-8)
Supplement: Supplementary file 1 — (DOCX 365 kb) [file 10389_2017_801_MOESM1_ESM.docx]

**Appendix 1: The estimated cost-effectiveness of NHS Health Checks**

Chang et al.[3] conducted a retrospective analysis of 138,788 patients aged 40–74 years registered with 462 English general practices participating in the Clinical Practice Research Datalink between 2009 and 2013, including those who had and had not attended the Health Checks. They applied a difference-in-difference methodology to compare changes in a number of outcomes between the two groups including mean BMI, with median follow up of 2 years. The authors found a small but statistically significant change in mean BMI -0.27 with a 95% CI of -0.20 to -0.34.

In order to incorporate this observed change in BMI into the EConDA tool, and thus determine the potential cost effectiveness of Health Checks through their impact on the level of obesity, it is necessary to convert the change in mean BMI into some estimate of reduced the proportion of obesity and overweight, the metric EConDA is set up to analyse interventions as (specifically percentage becoming a healthy weight from the overweight categories).

To do so an estimate of how the Chang estimated change in BMI equates to as a percentage of the population affected by the Health Checks moving to a healthy weight is needed. Using the relevant population statistics (40-74 year olds inclusive) drawn from the Health Survey for England (HSE) 2013 dataset it is possible to reproduce a mean change in observed BMI at a national level.

The current national BMI distribution from HSE for the relevant population is shown in Figure 2, combining both men and women (mean=28.2, SD=5.3, median=27.5). In the population eligible for Health Checks the proportion who are in the overweight or obese categories is 70.9% (i.e. BMI>=25.0), this is 3,004 in the HSE relevant population of 4,235.

Figure 2: Current BMI distribution in 40-74 year olds in England, HSE 2013

Applying the results from Forster require a number of assumptions, primarily:

1. The observed mean change is assumed to impact all of the population equally
2. The only impact of interest is the proportion of patients who move from the obese (>=30.0) and overweight (>=25.0) categories to the health weight (18.5 to 25.0 inclusive).
3. Patients who move from a healthy weight into the underweight category (BMI<18.5) are not considered in this analysis (which also overlooks the potential for Health Checks to move these patients into a healthy category by increasing their BMI in contrast to the primary aim of evaluating a reduction in BMI).

For a base case analysis we will use the weighted average of the BMI change observed in the Chang study of -0.27 across the entire population. Additional scenarios will consider the impact of using the BMI changes that would result if the upper and lower CI estimates estimated by Chang were observed (-0.20 and -0.34).

Applying the mean estimated change from Chang to the entire relevant population results in the distribution presented in Figure 3, as expected the change is almost unperceivable due to the small average change impacting the full population. As expected the mean BMI has reduced to 27.9 (SD=5.3, median=27.2). In this reduced BMI population 68.4% of the population is in the overweight or obese category, this is 2,896 in the HSE relevant population of 4,235. This shows the BMI change observed in Chang, if applied across the full population results in a reduction in the proportion of the overweight and obese category of 2.5% (70.9%-68.4%). This is equivalent to 3.5% of those who were overweight or obese before the Health Check becoming a healthy weight ((70.9%-68.4%)/70.9%).

Figure 3: Population BMI after Health Check impact (reduction of 0.27 points)

This percentage of those who were overweight or obese becoming a healthy weight from the above analysis (3.5%)^[[1]](#footnote-1)^ can be applied to the EConDA tool^[[2]](#footnote-2)^ alongside the estimates unit cost of a Health Check of £179^[[3]](#footnote-3)^ described in the main body of this paper. In addition to these settings a lifetime time horizon and a discount rate of 3.5% on both costs and outcomes was set in the EConDA tool. The ‘all risk’ cohort was selected in the tool to reflect the relevance of the Health Checks to the full population rather than a targeted ‘at risk’ population^[[4]](#footnote-4)^.

Figure 4 shows the impact of the intervention on the prevalence of each modelled disease over time. The figure shows a small absolute change in each of the disease incidence categories, with the largest being for diabetes where the difference between the two policy arms peaks at a difference of 1,276 cases per 100,000 population.

Figure 4: Estimated disease incidence over time from the EConDA tool


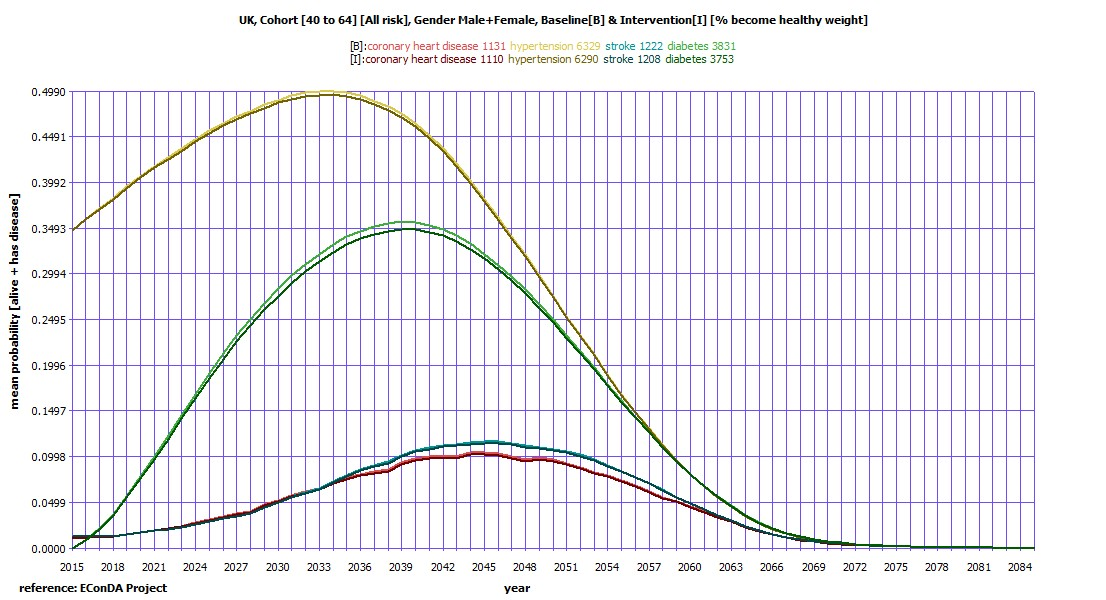


The EConDA output additionally allows us to estimate the cost-effectiveness of the Health Checks. As shown in Table 1 the EConDA tool estimates a life year gain of 0.05 years and QALY gain of 0.01 per patient. The cost of the Health Check and related interventions of £179 is set against the reduction in lifetime costs of the modelled diseases of £170, resulting in a small mean incremental lifetime per person cost of £9 per person. The small average health gain and cost combine to give an estimated incremental cost-effectiveness ratio (ICER) of £900/QALY. This results suggests that, under the assumptions of the base case analysis Health Checks are a highly cost-effective allocation of limited NHS resources when considered against a cost-effectiveness ratio of £20,000/QALY as applied by NICE.[7]

Table 1: Results of the analysis

|  | Baseline | With Health Checks | Increment |
| --- | --- | --- | --- |
| Life expectancy | 81.22 | 81.26 | 0.05 |
| Total QALYs | **55.51** | **55.52** | **0.01** |
| Cost of the Health Check | - | £179 | £179 |
| Disease cost | £13,356 | £13,186 | -£170 |
| Total costs | **£13,356** | **£13,412** | **£9** |
| **ICER** |  |  | **£900/QALY** |

A number of additional scenarios were also considered, varying the size of the change in BMI, and the population used in the EConDA tool. The results of these scenarios are presented in Table 2.

Table 2: Results of additional scenario analyses

|  | Incremental LY | Incremental QALYs | Incremental cost | ICER |
| --- | --- | --- | --- | --- |
| Baseline analysis (3.5% become healthy weight, 40-64 cohort, £179 cost of Checks) | 0.05 | 0.01 | £9 | £900/QALY |
| Lower CI estimate of BMI change (-0.20), 2.8% becoming healthy weight | 0.03 | 0.00 | £56 | Dominated |
| Upper CI estimate of BMI change (-0.34) 4.1% become healthy weight^[[5]](#footnote-5)^ | 0.05 | 0.01 | £9 | £900/QALY |
| 65+ cohort | 0.01 | 0.00 | £115 | Dominated |
| Cost of Checks increased to £302 | 0.05 | 0.01 | £132 | £13,200/QALY |

Table 2 highlights a number of important factors about this analysis. Firstly, the base case and upper estimated BMI change from Chang result in the same ICER. This is due to the rounding of the percentage of at risk patients who become a healthy weight to a integer level for implementation into the EConDA tool results in the same percentage change for the base case and lower CI analyses (with both 3.5% and 4.1% being rounded to 4%). Secondly, while the upper CI result is rounded to a 4% change for the toolkit, resulting in a different incremental life years estimate, the change is not enough to impact the incremental QALYs. In contrast the lower estimate from Change (mean BMI change of -0.20 points) results in a policy of Health Checks being dominated, that is more expensive but no more effective that a policy of no checks.

The change in the cohort age from 40-64 to 65+ results in no QALY benefit from the Health Checks. In addition, the cost saving from reduced disease costs estimated by EConDA is less than the base case cohort due to the reduction in remaining life over which the patients can benefit from a reduction in risk of disease due to reduction in BMI. As a result the intervention is dominated by current practice.

Finally, an additional potential source of an estimate of the cost of the Check and subsequent interventions was identified from the Impact Assessment produced by the Department of Health.[17] The Impact Assessment estimated an average annual cost of the Checks of £332 million (£302 per person receiving the Checks). This increased cost results in a substantial increase in the ICER to £13,200/QALY, still indicating cost-effectiveness despite the large relative increase in the cost estimate. It should be noted that the Impact Assessment annual cost estimate is not accompanied by additional evidence, and as such unlikely to be a better estimate of the cost of the Checks. In addition the Impact Assessment estimates an average annual net monetary benefit of £3,678 million, significantly more than the estimated annual cost.

The available evidence and use of the EConDA toolkit has necessitated several significant assumptions in addition to those outlined above and in previous literature by the authors of EConDA.[18] Primarily, the analysis assumes that the only beneficial impact of Health Checks on population health is through their ability to reduce obesity related disease by acting to reduce the BMI of the population. Additionally, the estimated cost of non-weight related follow-up interventions (such as smoking cessation) are included in the estimate of the cost of the Health Checks used in the analysis. This assumption should only act to bias against the cost-effectiveness of the Checks as they are unlikely to have a detrimental effect on other areas of health.

Secondly, the required rounding of the inputs and results of the EConDA tool have the potential to significantly impact the true ICER. For example as the QALY gain in the base case analysis is small (0.01) and the results from the tool only reported to two decimal placed it is theoretically possible that the true QALY gain is anywhere in the range of 0.005 to 0.015. These changes would result in a doubling of the ICER in the case of the former and a 50% decrease in the latter. This error is compounded by the rounding of the percentage who become a healthy weight to an integer value. However, even in the extreme case of a halving of the QALY gain to 0.005, as the incremental cost is so low in the base case the intervention remains significantly cost-effective (an ICER of £1,800/QALY).

Finally, due to the deterministic nature of the EConDA tool a full conceptualisation of the uncertainty is not possible. While the scenario analyses help to inform a number of areas of uncertainty a wide range of parametric uncertainty is not accounted for. Given the small QALY gains accrued over a long time horizon it is likely that while the mean base case result is highly cost-effective there exists a large level of uncertainty. However, as mentioned previously the low cost per person of the intervention coupled with its unobtrusive nature (implying no adverse events or mortality risks) may reduce the impact of this uncertainty in changing the overall decision of cost-effectiveness.

1. This has to be rounded to 4% for inputting into the EConDA model [↑](#footnote-ref-1)
2. The closest existing cohort in EConDA is 40 to 64 rather than to 74 as for the Health Checks [↑](#footnote-ref-2)
3. This requires manual estimation of the incremental cost conducted in this appendix as the EConDA tool only allows the inputting of annual costs rather than one off interventions. [↑](#footnote-ref-3)
4. ‘At risk’ is defined by the EConDA toolkit as those who are inactive individuals (<150 minutes of activity per week) [↑](#footnote-ref-4)
5. Same results as base case due to forced rounding of percentage become healthy weight for EConDA [↑](#footnote-ref-5)
